# Prevalence and socio-demographic correlates of tobacco and alcohol use in four sub-Saharan African countries: a cross-sectional study of middle-aged adults

**DOI:** 10.1186/s12889-021-11084-1

**Published:** 2021-06-12

**Authors:** Palwende Romuald Boua, Cassandra Claire Soo, Cornelius Debpuur, Innocent Maposa, Shai Nkoana, Shukri F. Mohamed, Solomon Choma, Abraham Oduro, Gershim Asiki, Lisa K. Micklesfield, Francesc Xavier Gómez-Olivé, Hermann Sorgho, Sumaya Mall, Michèle Ramsay, as members of AWI-Gen and the H3Africa Consortium

**Affiliations:** 1 Institut de Recherche en Sciences de la Santé, Clinical Research Unit of Nanoro, Nanoro, Burkina Faso; 2 Sydney Brenner Institute for Molecular Bioscience and Division of Human Genetics, Faculty of Health Sciences, University of the Witwatersrand, Johannesburg, South Africa; 3 Navrongo Health Research Centre, Biomedical Sciences Department, Navrongo, Ghana; 4 Division of Epidemiology and Biostatistics, School of Public Health, Faculty of Health Sciences, University of the Witwatersrand, Johannesburg, South Africa; 5 DIMAMO, Department of Pathology and Medical Sciences, School of Health Care Sciences, Faculty of Health Sciences, University of Limpopo, Polokwane, South Africa; 6 Health and Systems for Health Unit, African Population and Health Research Center (APHRC), Nairobi, Kenya; 7 MRC/Wits Developmental Pathways for Health Research Unit, Faculty of Health Sciences, University of the Witwatersrand, Johannesburg, South Africa; 8 MRC/Wits Rural Public Health and Health Transitions Research Unit, School of Public Health, Faculty of Health Sciences, University of the Witwatersrand, Johannesburg, South Africa

**Keywords:** Alcohol use, Tobacco use, Sub-Saharan Africa, Prevalence, Cross-sectional study, Adults, Socio-demographic correlates, AWI-gen

## Abstract

**Background:**

Substance misuse is a global public health problem. In addition to social and economic concerns, consumption of tobacco and alcohol is associated with susceptibility to cardiovascular, respiratory, and infectious diseases, cancers, and risk of transition to substance use disorders. African data suggest regional differences in the prevalence and patterns of substance use, but a number of key questions remain. This cross-sectional population-based study of middle-aged adults aims to examine prevalence and socio-demographic correlates of substance use in four sub-Saharan African countries, in rural and urban settings.

**Methods:**

Participants aged between 40 and 60 years were recruited from six research centres as part of the Africa Wits-INDEPTH partnership for Genomic Research study. Data on patterns of tobacco and alcohol consumption was captured, and the latter further assessed using the CAGE (cut-annoyed-guilty-eye) questionnaire.

**Results:**

Data from 10,703 participants suggested that more men (68.4%) than women (33.3%) were current substance users. The prevalence of current smoking was significantly higher in men than in women (34.5% vs 2.1%, *p* < 0.001). Smokeless tobacco was used more by women than men (14.4% vs 5.3%, p < 0.001). Current smoking was associated with alcohol consumption in men, and smoking cessation in men was associated with being a former drinker, having higher socio-economic status, and if married or cohabiting. Current alcohol consumption was higher in men, compared to women (60.3% vs 29.3%), and highest in men from Soweto (70.8%) and women from Nanoro (59.8%). The overall prevalence of problematic alcohol consumption among men was 18.9%, and women 7.3%. Men were significantly more likely to develop problematic drinking patterns, and this was more common in those who were divorced or widowed, and in current smokers.

**Conclusions:**

Regional variation in the patterns and prevalence of substance use was observed across study sites, and in rural and urban settings. The high levels of substance use recorded in this study are of concern due to the increased risk of associated morbidities. Further longitudinal data will be valuable in determining trends in substance misuse in Africa.

**Supplementary Information:**

The online version contains supplementary material available at 10.1186/s12889-021-11084-1.

## Background

Global data suggest that the prevalence of substance use (tobacco, alcohol, and recreational drugs) is increasing, especially in low and middle income countries (LMICs) [1–5]. On the African continent, there are few studies estimating prevalence and correlates of substance use, and data examining the socio-demographic correlates of substance use in African countries is sparse. Sreeramareddy and colleagues (2014) examined Demographic and Health Survey Data from 30 African countries to determine the prevalence and social correlates of smoking and smokeless tobacco [6]. Overall their findings suggest that the prevalence of smoking was higher in men than in women, and that tobacco use was more common among the poor, those with lower education, and working in occupations that required fewer skills. There are also studies that suggest that the prevalence of substance use varies regionally and between urban and rural settings, for example in Kenya, Ethiopia, and South Africa (SA) [3, 6–20]. Importantly, African studies examining the prevalence of substance use are not necessarily nationally representative and are often restricted to specific groups, such as among people living with HIV, homeless people, people suffering from mental disorders, or university students [3, 11, 15, 17, 19–22]. Socio-demographic correlates that have emerged from these studies include sex (i.e. men being more likely to engage in harmful substance use than women) and a range of physical and mental health outcomes associated with substance use [8, 10, 13, 17, 21, 23–26]. It is therefore important to examine prevalence and correlates of substance use, including sex, education, and area of residence (rural vs urban). Investigating these relationships may lead to a better understanding of the trajectory of substance use and pathways to various outcomes, and when best to intervene.

The Africa Wits-INDEPTH Partnership for Genomic Research (AWI-Gen) study was initiated in order to assess the genomic and environmental factors associated with susceptibility to cardiometabolic disease [27, 28]. Our secondary analysis addresses the paucity of data on prevalence and patterns of substance use in Africa, and examines demographic correlates of substance use in four sub-Saharan African (SSA) countries: South Africa (South), Kenya (East), Ghana and Burkina Faso (West). Our aim was to examine prevalence of substance use in rural and urban regions, including patterns of alcohol and tobacco consumption.

## Methods

### Study design and participants

The participants were recruited for the primary cross-sectional study, AWI-Gen, a Collaborative Centre of the Human Heredity and Health in Africa (H3Africa) Consortium, to examine genetic and environmental factors that contribute to cardiometabolic diseases in African populations [27, 28]. Participants were recruited among residents from six study sites in four SSA countries between 2013 and 2016. In SA, the sites were the MRC/Wits Agincourt Health and Demographic Surveillance System Site (HDSS) in Bushbuckridge (referred to as Agincourt), the Dikgale HDSS (now referred to as DIMAMO), and the Soweto cohort from the MRC/Wits Developmental Pathways for Health Research Unit [29–31]. In Kenya, representing East Africa, the study was conducted within two Nairobi slums by the African Population and Health Research Center (APHRC) HDSS in Nairobi [32]. In West Africa, in Ghana, at the Navrongo HDSS, Navrongo Health Research Centre, and Burkina Faso at the Nanoro HDSS, Institut de Recherche en Sciences de la Santé/ Clinical Research Unit of Nanoro [33, 34].

Participants of African descent aged between 40 and 60 years, and residing in the areas monitored by these sites were included in this cross-sectional population-based study in compliance with established recruitment procedures at each of the research centres [27–31, 33, 34]. Exclusion criteria included: closely related individuals, pregnant women, and non-residents [27, 28]. Participants were invited to complete a questionnaire requesting information on demography, family composition, marital status, education, employment, household attributes, substance use, and medical and biological variables not applicable to this study [28].

### Ethical considerations

All participants provided written informed consent prior to enrollment into the study [28]. Ethics clearance for each of the study sites was approved through their relevant institutional and national ethics boards, and the AWI-Gen study as a whole was approved by the Human Research Ethics Committee (HREC) (Medical) of the University of the Witwatersrand (approval number M121029, and renewal M170880). Participant identity was protected by assigning unique study identifiers and the key linking them was securely stored at the collection site.

### Measures

Overall substance use was defined as; ‘current substance user’ versus ‘current non-user’ (a user was defined as a person currently consuming alcohol and/or currently consuming tobacco products (smoking, chewing, or using snuff)).

Tobacco use variables included smoking categorised as; never used, current user, or former user, based on participant responses. Age at smoking initiation and type of tobacco smoked (cigarettes, pipe, hand rolled cigarettes, and cigars - note that participants could report more than one) were also recorded. The frequency of smoking was captured as; daily (5–6 days per week), 1–4 days per week, 1–3 days per month, or less than once per month. All participants were asked if they used smokeless tobacco and if so; was it chewing tobacco, or snuff.

Alcohol use was categorised as; never consumed, current non-problematic consumer, current problematic consumer, or former consumer. Problematic alcohol use was determined according to the CAGE (cut-annoyed-guilty-eye) questionnaire [35]. If the participant responded yes to at least two of the following questions they were categorised as being problematic drinkers: Have you ever felt that you should cut down on your drinking? Have people annoyed you by criticising your drinking? Have you ever felt bad or guilty about your drinking? Have you ever had an alcoholic drink first thing in the morning to steady your nerves, or get rid of a hangover? In the past year, did you ever have 6 or more alcoholic drinks in a single morning, afternoon, or night? Data were collected on the type of alcohol consumed (spirits, beer, homebrew, wine, or other) and the frequency of alcohol consumption (daily, 5–6 days per week, 1–4 days per week, 1–3 days per month, or less than once per month). It is important to point out that ‘Other’ for the type of alcohol refers to locally brewed alcohol ranging from home brewed sorghum beer in Nanoro and Navrongo, Changaa (traditional home-brewed spirit from maize, millet, or sorghum) in Nairobi, to fermented cider in Dikgale, and cider and spirits in Soweto.

Household goods were used as a proxy for socioeconomic status (SES), using a method developed by the Demographic and Health Surveys (DHS) Program [36]. This method involves a principal component analysis of the SES variables (household attributes) where factor scores (factor loadings) are used to predict wealth indices (from the first principal component or factor) which in turn are categorised into quintiles [37]. The partnership status of participants was categorised as; never married/co-habited, married/living with partner, or divorced/widowed. Education was reported as no formal education, primary, secondary, or tertiary education. Employment status was considered as either employed, or unemployed.

### Statistical analyses

Data were captured into REDCap and underwent basic quality control (QC) [38]. Further QC was completed in a PostgreSQL database after exportation. Descriptive statistics were used to summarise the socio-demographic variables in current substance users and non-users of alcohol and/or tobacco, and the distributions were compared. Continuous variables were reported as medians and inter-quartile ranges, and categorical variables were reported as percentages. The data on the consumption of alcohol and tobacco were analysed separately for each site according to sex, and for the combined dataset. The Kruskal-Wallis test was used to compare continuous variables between the sites. Two group comparisons for continuous and non-normally distributed variables were performed using the Mann-Whitney test, and Pearson Chi-squared test or Fisher’s exact test for categorical variables to assess differences between the sexes.

To assess the correlates with alcohol use of the participants according to current use (current consumer of tobacco and/or alcohol) or current non-use (individuals who never consumed or are former consumers of tobacco and/or alcohol), abuse (problematic alcohol use vs non-problematic alcohol use), and smoking cessation (for men); multivariable logistic regression was used to calculate adjusted Odds Ratios (aORs) with 95% Confidence Intervals. We adjusted for ethnicity based on recruitment site for all logistic regressions. Some analyses were performed only for men because the number of women who smoked tobacco was negligible. The analyses were performed using Stata 14.2© (45).

## Results

### Participants: description of socio-demographic variables in current users and current non-users of tobacco and alcohol

The study included 10,703 participants aged between 40 and 60 years, of whom 5895 were women (55.1%). Table 1 presents the characteristics of the participants according to current use (current consumer of tobacco and/or alcohol) or current non-use (individuals who never consumed or are former consumer of tobacco and/or alcohol), stratified by study site, and for the combined sample. The overall median age was 50 years. In the combined dataset, age was significantly associated with substance use, although age was not significant in four of the six study sites. Similarly level of education was significantly associated with substance use in the combined dataset, although the distribution of education levels differed across sites and was only significantly associated with substance use in three of the six sites. Overall, there were more substance users among those with no formal education. SES (assessed using quintiles) showed differences in the distribution between substance users and non-users across sites. Substance use was variable across SES quintiles, and significantly associated across all study sites. There were more substance users among participants who had never married/cohabited or married/living with partner, than among those who were divorced/widowed. More employed participants were substance users than those that were unemployed. Complete data regarding substance use was not available for Soweto, as alcohol consumption was not recorded for Soweto women.

**Table 1:** Socio-demographic characteristics of the AWI-Gen cohort according to study site and for the combined dataset (All sites), stratified according to substance use (current alcohol consumption and/or current tobacco use, including smoking, chewing, and using snuff)

	Agincourt			Digkale			Nairobi			Nanoro			Navrongo			Soweto			All sites		
	Current users	Current non-users	*p*-value	Current users	Current non-users	p-value	Current users	Current non-users	p-value	Current users	Current non-users	p-value	Current users	Current non-users	p-value	Current users	Current non-users	p-value	Current users	Current non-users	p-value
**N(%)**	358 (24.40%)	1107 (75.60%)		655 (43.90%)	512 (56.10%)		485 (25.00%)	1457 (75.00%)		1467 (70.40%)	617 (29.60%)		1410 (70.00%)	604 (30.00%)		872 (42.90%)	1159 (57.00%)		5247 (49.00%)	5456 (51.00%)	
**Age (years)**	52 (10)	51 (10)	0.1782	51 (10)	40.0 (10.5)	**< 0.001**	47 (9)	48 (9)	0.8415	50 (10)	49 (10)	**< 0.001**	51 (10)	51 (10)	0.3125	49 (10)	49 (10)	0.4236	50 (10)	49 (10)	**< 0.001**
**Sex**			**< 0.001**			**< 0.001**			**< 0.001**			0.674			**< 0.001**			**< 0.001**			**< 0.001**
Women	85 (9.53%)	807 (90.47%)		380 (46.86%)	431 (53.14%)		91 (8.62%)	965 (91.38%)		727 (63.97%)	312 (30.03%)		628 (57.56%)	463 (42.44)%		49 (4.87%)^a^	957 (95.13%)^a^		1960 (33.25%)	3935 (66.75%)	
Men	273 (47.64%)	300 (52.36%)		275 (77.25%)	81 (22.75%)		394 (44.47%)	492 (55.53%)		741 (70.81%)	305 (29.19%)		782 (84.72%)	141 (15.28%)		823 (80.29%)	202 (19.71%)		3287 (68.37%)	1521 (31.63%)	
**Socioeconomic Status (Lowest to highest)**			**< 0.001**			**0.025**			**0.023**			**< 0.001**			**0.023**			**< 0.001**			**< 0.001**
1st Quintile	82 (36.28%)	144 (63.72%)		93 (63.27%)	54 (36.73%)		78 (33.19%)	157 (66.81%)		246 (73.21%)	90 (26.79%)		243 (65.32%)	129 (34.68%)		36 (15.52%)	196 (84.48%)		778 (50.26%)	770 (49.74%)	
2nd Quintile	103 (29.68%)	244 (70.32%)		155 (58.49%)	110 (41.51%)		107 (24.77%)	325 (75.23%)		303 (75.00%)	101 (25.00%)		250 (69.44%)	110 (30.56%)		100 (20.49%)	388 (79.51%)		1018 (44.34%)	1278 (55.66%)	
3rd Quintile	42 (22.95%)	141 (77.05%)		89 (58.17%)	64 (41.83%)		112 (24.89%)	338 (75.11%)		303 (74.81%)	102 (25.19%)		288 (74.42%)	99 (25.58%)		132 (40.87%)	191 (59.13%)		966 (50.82%)	935 (49.18%)	
4th Quintile	80 (22.99%)	268 (77.01%)		148 (58.04%)	107 (41.96%)		85 (21.46%)	311 (78.54%)		282 (73.06%)	104 (26.94%)		346 (73.15%)	127 (26.85%)		221 (63.51%)	127 (36.49%)		1162 (52.67%)	1044 (47.33%)	
5th Quintile	51 (14.13%)	310 (85.87%)		170 (49.13%)	176 (50.87%)		103 (24.01%)	326 (75.99%)		331 (60.62%)	215 (39.38%)		283 (67.22%)	138 (32.78%)		373 (76.59%)	114 (23.41%)		1311 (50.62%)	1279 (49.38%)	
**Marital status**			**< 0.001**			**0.016**			**< 0.001**			0.140			**< 0.001**			**< 0.001**			**< 0.001**
Never married or co-habited	49 (36.57%)	85 (63.43%)		156 (54.17%)	132 (45.83%)		13 (15.66%)	70 (84.34%)		10 (58.82%)	7 (41.18%)		16 (80.00%)	4 (20.00%)		239 (75.63%)	77 (24.37%)		483 (56.29%)	375 (43.71%)	
Married/living with partner	252 (25.66%)	730 (74.34%)		324 (53.64%)	280 (46.36%)		374 (28.90%)	920 (71.10%)		1271 (70.03%)	544 (29.97%)		1067 (72.05%)	414 (27.95%)		446 (53.35%)	390 (46.65%)		3734 (53.25%)	3278 (46.75%)	
Divorced/Widowed	57 (16.33%)	292 (83.67%)		175 (63.64%)	100 (36.36%)		98 (17.38%)	466 (82.62%)		185 (75.20%)	61 (24.80%)		327 (63.87%)	185 (36.13%)		165 (30.78%)	371 (69.22%)		1007 (40.57%)	1475 (59.43%)	
**Highest education completed**			0.060			**< 0.001**			**0.006**			0.086			0.403			**< 0.001**			**< 0.001**
No Formal education	106 (26.30%)	297 (73.70%)		63 (65.62%)	33 (34.38%)		25 (17.01%)	122 (82.99%)		1229 (71.54%)	489 (28.46%)		980 (69.36%)	433 (30.64%)		7 (70.00%)	3 (30.00%)		2410 (63.64%)	1377 (36.36%)	
Primary	148 (25.74%)	427 (74.26%)		250 (64.77%)	136 (35.23%)		263 (23.69%)	847 (76.31%)		157 (65.69%)	82 (34.31%)		282 (73.63%)	101 (26.37%)		122 (16.18%)	632 (83.82%)		1222 (35.45%)	2225 (64.55%)	
Secondary	91 (22.86%)	307 (77.14%)		328 (51.01%)	315 (48.99%)		187 (28.38%)	472 (71.62%)		62 (64.58%)	34 (35.42%)		121 (69.14%)	54 (30.86%)		620 (69.27%)	275 (30.73%)		1409 (49.16%)	1457 (50.84%)	
Tertiary	12 (13.64%)	76 (86.36%)		14 (34.15%)	27 (65.85%)		10 (38.46%)	16 (61.54%)		15 (83.33%)	3 (16.67%)		24 (66.67%)	12 (33.33%)		106 (69.28%)	47 (30.72%)		181 (50.00%)	181 (50.00%)	
**Employment status**			0.700			0.259			0.168			0.380			**< 0.001**			**0.018**			**< 0.001**
Unemployed	215 (24.32%)	669 (75.68%)		396 (54.70%)	328 (45.30%)		22 (19.47%)	91 (80.53%)		13 (61.90%)	8 (38.10%)		564 (75.20%)	186 (24.80%)		322 (39.75%)	488 (60.25%)		1532 (46.40%)	1770 (53.60%)	
Employed	117 (23.40%)	383 (76.60$)		255 (58.09%)	184 (41.91%)		461 (25.25%)	1365 (74.75%)		1453 (70.67%)	603 (29.33%)		842 (66.93%)	416 (33.07%)		550 (45.05%)	671 (54.95%)		3678 (50.38%)	3622 (49.62%)	

Continuous variables are presented as medians and interquartile range. Mann-Whitney Wilcoxon test has been used to assess differences

Categorical variables are presented as number and percentage. Chi-squared or Fisher’s exact test (when Chi-squared test conditions were not fulfilled) were used to assess differences

^a^This only accounts for smoking since alcohol consumption data have not been collected for women in Soweto

### Tobacco use

The prevalence of current smoking was significantly higher in men than in women (34.5% vs 2.1%, *p* < 0.001). There were differences observed between the sites with the lowest prevalence of current smoking in Nanoro for both men and women (13.6 and 0% respectively), and the highest in Dikgale for men (63.4%) and in Soweto for women (4.9%) (Table 2). The median age of smoking initiation was 20 (Q1-Q3: 17–24) years old for both sexes. Cigarettes were the most consumed type of smoking tobacco, followed by hand-rolled cigarettes and pipes. For men, Nairobi had the highest prevalence of former smokers (29.3%), and Nanoro had the lowest percentage of former smokers (11.7%). Smokeless tobacco was used more by women overall (14.4% vs 5.3%, *p* < 0.001), and chewing tobacco (7.1%) more than snuff (5.4%) for women across all sites. The type and frequency of smoked tobacco was not available for Agincourt and Soweto, and smokeless tobacco data was unavailable for Soweto.

**Table 2:** Prevalence and characteristics of tobacco consumption per site and per gender

	Agincourt			Digkale			Nairobi			Nanoro			Navrongo			Soweto			All men	All women	
	Men (573)	Women (892)	p-value	Men (355)	Women (810)	p-value	Men (886)	Women (1056)	p-value	Men (1045)	Women (1039)	p-value	Men (923)	Women (1091)	p-value	Men (1025)	Women (1005)	p-value	*N* = 4807	*N* = 5893	p-value
**Smoking status**			**< 0.001**			**< 0.001**			**< 0.001**			**< 0.001**			**< 0.001**			**< 0.001**			**< 0.001**
Never Smoked	291 (50.96%)	882 (98.88%)		54 (15.21%)	756 (93.33%)		418 (47.33%)	975 (92.33%)		779 (74.69%)	1033 (99.81%)		332 (35.97%)	1052 (96.6%)		311 (30.40%)	897 (89.97%)		2185 (45.52%)	5595 (95.17%)	
Current smoker	155 (27.15%)	3 (0.34%)		224 (63.38%)	25 (03.09%)		208 (23.50%)	27 (02.56%)		142 (13.61%)	0 (0.00%)		388 (42.04%)	21 (01.93%)		540 (52.79%)	49 (4.91%)		1658 (34.54%)	125 (02.13%)	
Former smoke	125 (21.89%)	7 (0.78%)		76 (21.41%)	29 (03.58%)		259 (29.27%)	54 (05.11%)		122 (11.70%)	2 (0.19%)		203 (21.99%)	16 (01.47%)		172 (16.81%)	51 (5.12%)		957 (19.94%)	159 (02.70%)	
**Age started smoking**	20 (17–27)	23 (20–30)	0.4103	19 (16–23)	19 (16–22)	0.7572	19 (16–22)	20 (17–23)	0.1457	20 (18–25)	24.5 (21–28)	0.3333	20 (20–25)	20 (18–30)	0.8061	NA	NA		20 (17–25)	20 (17–24)	0.3125
**Type of smoked tobacco Use**																					
Cigarettes (Yes)	NA	NA	**–**	118 (33.15%)	14 (01.73%)	**< 0.001**	208 (23.48%)	25 (02.37%)	**< 0.001**	140 (13.40%)	0 (0.00%)	**< 0.001**	262 (28.39%)	12 (01.10%)	**< 0.001**	535 (52.20%)	49 (04.87%)	**< 0.001**	1263 (26.27%)	100 (01.70%)	**< 0.001**
Pipe (Yes)	NA	NA	**–**	39 (10.96%)	5 (0.62%)	**< 0.001**	0 (0.00%)	1 (0.09%)	0.360	1 (0.10%)	0 (0.00%)	0.321	5 (0.54%)	1 (0.09%)	0.034	3 (0.29%)	0 (0.00%)	0.086	48 (01.00%)	7 (0.12%)	**< 0.001**
Hand rolled (Yes)	NA	NA		77 (21.63%)	6 (0.74%)	**< 0.001**	0 (0.00%)	1 (0.09%)	0.360	0 (0.00%)	0 (0.00%)		164 (17.77%)	11 (1.01%)	**< 0.001**	NA	NA		241 (05.01%)	18 (0.31%)	**< 0.001**
Cigars (Yes)	NA	NA	**–**	0 (0.00%)	0 (0.00%)		0 (0.00%)	0 (0.00%)		0 (0.00%)	0 (0.00%)		2 (0.22%)	0 (0.00%)	0.124	NA	NA		2 (0.04%)	0 (0.00%)	
**Frequency of smoked tobacco use**						**< 0.001**			**< 0.001**			**< 0.001**			**< 0.001**						**< 0.001**
Current Non-smokers				131 (36.8%)	786 (96.92%)		678 (76.52%)	1029 (97.44%)		903 (86.41%)	1039 (100%)		535 (57.96%)	1070 (98.08%)		NA	NA		2665 (70.45%)	4813 (98.45%)	
Daily smokers	NA	NA		194 (54.49%)	20 (02.47%)		182 (20.54%)	21 (01.99%)		126 (12.06%)	0 (0.00%)		343 (37.16%)	18 (01.65%)		NA	NA		845 (22.34%)	59 (01.21%)	
5–6 days per week	NA	NA		3 (0.84%)	0 (0.00%)		2 (0.23%)	0 (0.00%)		7 (0.67%)	0 (0.00%)		17 (01.84%)	0 (0.00%)		NA	NA		29 (0.77%)	0 (0.00%)	
1–4 days per week	NA	NA		23 (06.46%)	4 (0.49%)		21 (02.37%)	6 (0.57%)		6 (0.57%)	0 (0.00%)		17 (01.84%)	0 (0.00%)		NA	NA		67 (01.77%)	10 (0.20%)	
1–3 days per month	NA	NA		4 (1.12%)	1 (0.12%)		0 (0.00%)	0 (0.00%)		2 (0.19%)	0 (0.00%)		3 (0.33%)	1 (0.09%)		NA	NA		9 (0.24%)	2 (0.04%)	
Less than once per month	NA	NA		1 (0.28%)	0 (0.00%)		3 (0.34%)	0 (0.00%)		0 (0.00%)	0 (0.00%)		0 (0.00%)	0 (0.00%)		NA	NA		4 (0.11%)	0 (0.00%)	
**Smokeless tobacco status (Yes)**	13 (02.27%)	51 (05.72%)	**0.002**	32 (08.99%)	337 (41.55%)	**< 0.001**	48 (05.42%)	20 (01.89%)	**< 0.001**	67 (06.41%)	326 (31.38%)	**< 0.001**	95 (10.29%)	114 (10.45%)	0.735	NA	NA		255 (05.30%)	848 (14.39%)	**< 0.001**
**Type of smokeless tobacco Use**																					
Snuff (Yes)	4 (0.70%)	31 (03.48%)	**0.002**	18 (05.06%)	253 (31.20%)	**< 0.001**	35 (03.95%)	16 (01.52%)	**< 0.001**	3 (0.29%)	4 (0.38%)	**< 0.001**	30 (03.25%)	12 (01.10%)	**0.002**	0 (0.00%)	0 (0.00%)	**–**	90 (01.87%)	316 (05.36%)	**< 0.001**
Chewing tobacco use (Yes)	0 (0.00%)	0 (0.00%)	**–**	0 (0.00%)	12 (01.48%)	**< 0.001**	4 (0.45%)	4 (0.38%)	**< 0.001**	64 (06.12%)	307 (29.55%)	**< 0.001**	58 (06.28%)	93 (08.52%)	**< 0.001**	0 (0.00%)	0 (0.00%)	**–**	126 (02.62%)	416 (07.06%)	**< 0.001**

Categorical variables are presented as number and percentage. Chi-squared or Fisher’s exact test (when Chi-squared test conditions were not fulfilled) were used to assess differences

*NA* For variables not available at site

### Alcohol consumption

The patterns of alcohol consumption per site, and for the combined sample (All) are presented in Table 3. In the combined sample, the percentage of lifetime abstainers (never consumed) was more than twofold higher among women than men (52.4% vs 23.8%). Overall, current consumers were more likely to be men. The lowest prevalence of current alcohol consumption was observed in Nairobi (33.9% of men and 5.9% of women). Consumers of alcohol were highest in Soweto (70.8%) for men, and in Nanoro (59.8%) for women. Navrongo had the highest rate of problematic drinking (50.1% men and 15.3% women). Former consumers were highest in Nairobi (37.1%) for men, and Navrongo for women (24.3%). Among the sites, Nanoro had the highest prevalence of daily alcohol consumption, whilst Dikgale had the lowest. There were differences in the types of alcoholic beverages consumed at each site, despite homebrewed alcohol and beer being most popular in most sites (Table 3). Approximately 21.7% of men felt that they should cut down on alcohol consumption, and 8.6% of women felt the same. Alcohol consumption-based guilt was felt by 16.9% of men and 5.1% of women. Binge drinking (> 6 alcoholic drinks) was observed at a higher prevalence in men than in women (9.6% vs 2.5%, *p* < 0.001) (Table 3). Data on alcohol consumption was not collected for Soweto women, nor was the frequency of consumption and CAGE questionnaire responses available for Soweto men.

**Table 3:** Prevalence and characteristics of alcohol consumption per site and per gender

	Agincourt			Digkale			Nairobi			Nanoro			Navrongo			Soweto			All Men	All Women	
	Men (573)	Women (892)	p-value	Men (355)	Women (810)	p-value	Men (886)	Women (1056)	*p*-value	Men (1045)	Women (1039)	p-value	Men (923)	Women (1091)	p-value	Men (1025)	Women (1005)	p-value	N = 4807	N = 5893	p-value
**Alcohol use**			**< 0.001**			**< 0.001**			**< 0.001**			**< 0.001**			**< 0.001**						**< 0.001**
**Never Consumed**	187 (32.64%)	731 (81.95%)		55 (15.45%)	561 (69.43%)		257 (29.01%)	758 (71.78%)		273 (26.22%)	277 (26.74%)		71 (7.71%)	231 (21.21%)		299 (29.17%)	–		1142 (23.79%)	2558 (52.41%)	
**Current non-problematic**	218 (38.08%)	52 (05.83%)		77 (21.63%)	45 (05.57%)		174 (19.64%)	32 (03.03%)		544 (52.26%)	521 (50.19%)		252 (27.36%)	426 (39.12%)		726 (70.83%)	–		1991 (41.97%)	1075 (22.02%)	
**Current problematic**	18 (03.14%)	2 (0.22%)		142 (39.89%)	57 (07.05%)		126 (14.22%)	30 (02.84%)		160 (15.37%)	100 (09.65%)		461 (50.05%)	167 (15.34%)		–	–		906 (18.87%)	356 (07.29%)	
**Former consumer**	150 (26.18%)	107 (12.00%)		82 (23.03%)	145 (17.95%)		329 (37.13%)	236 (22.35%)		64 (06.15%)	139 (13.42%)		137 (14.88%)	265 (24.33%)		–	–		762 (15.87%)	892 (18.27%)	
**Problems with drinking**																					
**Felt should cut down?**	17 (02.97%)	2 (0.22%)	**< 0.001**	140 (39.33%)	65 (08.01%)	**< 0.001**	137 (15.46%)	31 (02.94%)	**< 0.001**	208 (19.90%)	126 (12.13%)	**< 0.001**	543 (58.83%)	282 (25.85%)	**< 0.001**	NA	NA		1045 (21.73%)	506 (08.58%)	**< 0.001**
**People have criticized**	8 (01.40%)	1 (0.11%)	**0.002**	90 (25.28%)	33 (04.07%)	**< 0.001**	95 (10.72%)	27 (02.56%)	**< 0.001**	136 (13.01%)	94 (09.05%)	**0.010**	347 (37.59%)	117 (10.72%)	**< 0.001**	NA	NA		676 (14.06%)	272 (04.62%)	**< 0.001**
**Guilty?**	10 (01.75%)	1 (0.11%)	**< 0.001**	121 (33.99%)	54 (06.66%)	**< 0.001**	127 (14.33%)	27 (02.56%)	**< 0.001**	111 (10.62%)	51 (04.91%)	**< 0.001**	441 (47.78%)	170 (15.58%)	**< 0.001**	NA	NA		810 (16.85%)	303 (05.14%)	**< 0.001**
**Alcohol first in the morning?**	30 (05.24%)	3 (0.34%)	**< 0.001**	121 (33.99%)	20 (02.47%)	**< 0.001**	51 (05.76%)	10 (0.95%)	**< 0.001**	87 (08.33%)	63 (06.06%)	**0.136**	229 (24.81%)	49 (04.49%)	**< 0.001**	NA	NA		518 (10.77%)	145 (02.46%)	**< 0.001**
**> 6 alcoholic drinks**	98 (17.10)	11 (01.23%)	**< 0.001**	136 (38.20%)	49 (06.04%)	**< 0.001**	43 (04.85%)	9 (0.85%)	**< 0.001**	166 (15.89%)	74 (07.12%)	**< 0.001**	18 (01.95%)	5 (0.46%)	**0.007**	NA	NA		461 (09.59%)	148 (02.51%)	**< 0.001**
**Type of consumption**																					
**Beer**	139 (24.26%)	24 (02.69%)	**< 0.001**	217 (60.96%)	98 (12.08%)	**< 0.001**	334 (37.70%)	190 (17.99%)	**< 0.001**	154 (14.74%)	13 (01.25%)	**< 0.001**	250 (27.09%)	158 (14.48%)	**< 0.001**	593 (57.85%)	NA		1687 (35.09%)	483 (08.19%)	**< 0.001**
**Home brew**	85 (14.83%)	22 (02.47%)	**< 0.001**	73 (20.51%)	34 (04.19%)	**< 0.001**	361 (40.74%)	120 (11.36%)	**< 0.001**	223 (21.34%)	393 (37.82%)	**< 0.001**	675 (73.13%)	772 (70.76%)	**< 0.001**	25 (02.44%)	NA		1442 (29.99%)	1341 (22.75%)	**< 0.001**
**Spirits**	7 (01.22%)	1 (0.11%)	**< 0.001**	3 (0.85%)	2 (0.25%)	**< 0.001**	25 (02.82%)	7 (0.66%)	**< 0.001**	60 (05.74%)	7 (0.67%)	**< 0.001**	629 (68.15%)	391 (35.84%)	**< 0.001**	59 (05.76%)	NA		783 (16.29%)	408 (06.92%)	**< 0.001**
**Wine**	3 (0.52%)	3 (0.34%)	**< 0.001**	2 (0.56%)	25 (03.08%)	**< 0.001**	5 (0.56%)	4 (0.38%)	**< 0.001**	5 (0.48%)	7 (0.67%)	0.803	33 (03.58%)	11 (01.01%)	**< 0.001**	41 (04.00%)	NA		89 (01.85%)	50 (0.85%)	**< 0.001**
**Other**	1 (0.17%)	4 (0.45%)	0.380	11 (03.10%)	87 (10.74%)	**< 0.001**	1 (0.11%)	0 (0.00%)	0.275	411 (39.33%)	345 (33.21%)	**0.007**	4 (0.43%)	3 (0.27%)	**< 0.001**	54 (05.27%)	NA		482 (10.02%)	439 (07.45%)	**< 0.001**
**Frequency of consumption**			**< 0.001**			**< 0.001**			**< 0.001**			**< 0.001**			**< 0.001**						**< 0.001**
**Current Non-consumers**	337 (58.81%)	838 (93.95%)		137 (38.59%)	710 (87.65%)		588 (66.37%)	994 (94.13%)		345 (33.01%)	423 (40.71%)		211 (22.86%)	506 (46.38%)		NA	NA		2643 (54.98%)	4476 (75.95%)	
**Daily**	37 (06.46%)	4 (0.45%)		12 (03.38%)	0 (0.00%)		78 (08.80%)	12 (01.14%)		284 (27.18%)	107 (10.30%)		125 (13.54%)	60 (05.50%)		NA	NA		536 (11.15%)	183 (03.11%)	
**5–6 days per week**	35 (06.11%)	3 (0.34%)		7 (01.97%)	0 (0.00%)		6 (0.68%)	1 (0.09%)		97 (09.28%)	44 (04.23%)		263 (28.39%)	128 (11.73%)		NA	NA		407 (08.47%)	176 (02.99%)	
**1–4 days per week**	66 (11.52%)	7 (0.78%)		137 (38.59%)	35 (04.32%)		129 (14.56%)	28 (02.65%)		268 (25.65%)	354 (34.07%)		272 (29.47%)	312 (28.60%)		NA	NA		872 (18.14%)	736 (12.49%)	
**1–3 days per month**	60 (10.47%)	18 (02.02%)		61 (17.18%)	59 (07.28%)		58 (06.55%)	12 (01.14%)		43 (04.11%)	93 (08.95%)		43 (04.66%)	65 (05.96%)		NA	NA		265 (05.51%)	247 (04.19%)	
**Less than once per month**	38 (06.63%)	22 (02.47%)		1 (0.28%)	6 (0.74%)		27 (03.05%)	9 (0.85%)		8 (0.77%)	18 (01.73%)		10 (01.08%)	20 (01.83%)		NA	NA		84 (01.75%)	75 (01.27%)	

Categorical variables are presented as number and percentage. Chi-squared or Fisher’s exact test (when Chi-squared test conditions were not fulfilled) were used to assess differences

*NA* Variables not available at site

### Multivariable logistic regression analysis of problematic drinking

**Fig. 1:**
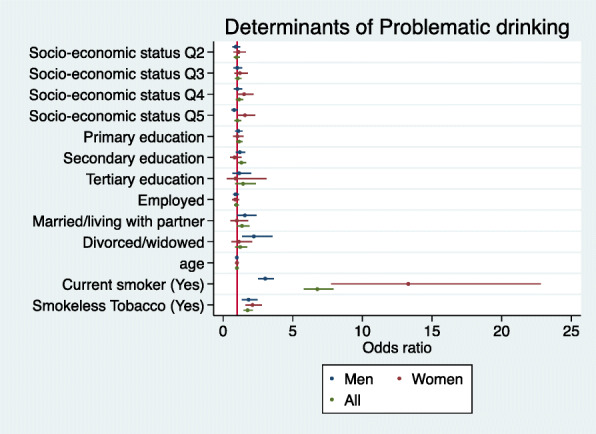
Forest plot depicting the aORs for the determinants of problematic alcohol consumption in men and women for the combined dataset. (Legend) Forest plot of multivariable logistic regression coefficients (95% Confidence Intervals) for the association of problematic drinking with socio-economic status (SES quintiles) (Reference = Q1), highest education level (Reference = No formal education), sex (Reference = Women), employment status (Reference = Not employed), marital status (Reference = Single), age, smoking status (Reference = current non-smokers), smokeless tobacco use (Reference = smokeless tobacco non-user). The model adjusted for site as a proxy for ethnicity based on location of research centre (*N* = 8486)

In the site (proxy for ethnicity) adjusted analysis; SES, age, level of education, and employment status were not associated with problematic alcohol use when pooling data from all of the sites (Fig. 1; Supplementary Table 1). Being male, and being divorced/widowed was associated with increased likelihood of alcohol abuse with respective aOR of 3.23 (95% IC [2.72–3.82]) and 1.61 (95% IC [1.12–2.32]) (Supplementary Table 1). These associations differed at site level, and being male was the only consistent risk factor across all sites with aOR ranging from 1.91 (95% IC [1.36–2.69]) in Nanoro, to 8.15 (95% IC [1.34–49.66]) in Agincourt. Higher SES was associated with reduced odds in Nairobi, whereas in Nanoro it was associated with increased odds of having a drinking problem. When assessing the effects of tobacco consumption on problematic drinking, tobacco users showed increased likelihood of problematic alcohol consumption with aOR = 3.84 (95% IC [3.24–4.57]) for current smokers, and aOR = 2.07 (95% IC [1.70–2.52]) for smokeless tobacco users.

### Analysis of current smoking in men

**Fig. 2:**
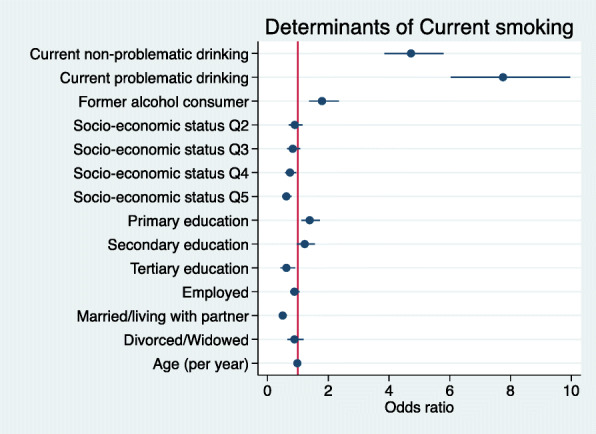
Forest plot showing the correlates of current tobacco smoking for men only. (Legend) Forest plot of regression coefficients (95% Confidence Intervals) for the association of current smoking in men with socio-economic status (SES quintiles) (Reference = Q1), highest level of education (Reference = No formal education), employment (Reference = Not employed), marital status (Reference = Single), age, alcohol consumption (Reference = never consumed). Model adjusted for site as a proxy for ethnicity. Estimates for men only (*N* = 4734)

There were too few women smokers for meaningful analysis, so only men were included in this analysis. Multivariable logistic regression suggests that SES (4th and 5th quintiles), being married/living with partner, and tertiary education were significantly associated with a reduced likelihood of being a current smoker (Fig. 2). In men, alcohol consumption and having only primary school education were associated with statistically increased odds of being a current smoker, while age was associated with reduced likelihood of being a current smoker, which although statistically significant had a small effect in our cohort of older adults (Fig. 2, Supplementary Table 2).

### Correlates of smokeless tobacco use

**Fig. 3:**
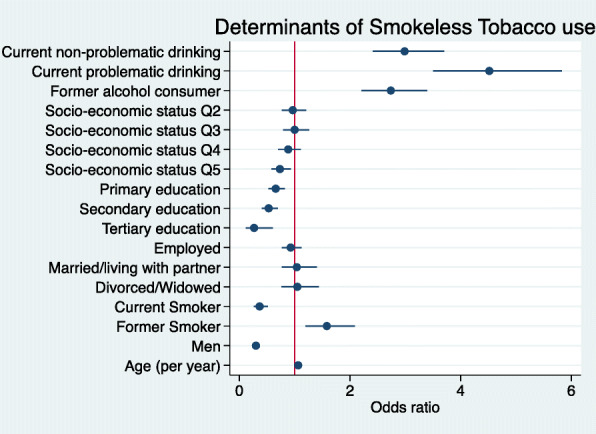
Forest plot indicating the site aORs for the correlates of smokeless tobacco consumption. (Legend) Forest plot of regression coefficients (95% Confidence Intervals) for the association of smokeless tobacco use with socio-economic status (SES quintiles) (Reference = Q1), highest level of education (Reference = No formal education), employment (Reference = Not employed), marital status (Reference = Single), age, sex (Reference = Women), alcohol consumption (Reference = never consumed), smoking status (Reference = Never smoked). Model adjusted for site as a proxy for ethnicity. (*N* = 8485)

Overall results across all sites suggest that smokeless tobacco use was more likely if the participant consumed alcohol (current non-problematic alcohol consumer aOR = 2.99 95% CI [2.41–3.70]; current problematic aOR = 4.52 [3.50–5.83]; former consumer aOR = 2.74 [2.21–3.39]) or were former smokers (aOR = 1.58, 95% IC [1.20–2.09]) (Fig. 3, Supplementary Table 3). On the other hand, current smokers were less likely to use smokeless tobacco (aOR = 0.37, 95% IC [0.26–0.51]) and men were significantly less likely to use smokeless tobacco (aOR = 0.30, 95% IC [0.24–0.37]). Figure 3 provides a graphic summary of the results from the multivariable logistic regression performed.

### Multivariable logistic regression analysis of smoking cessation in men

**Fig. 4:**
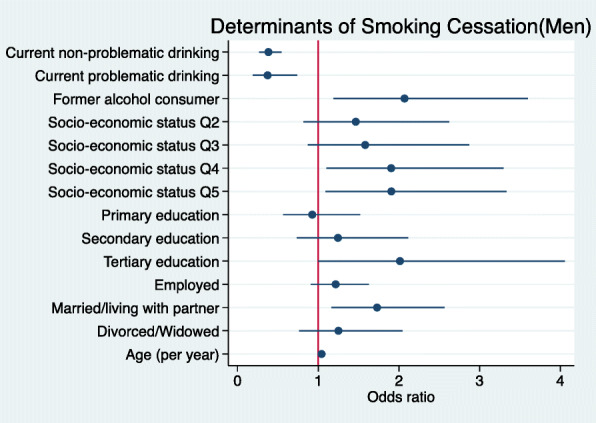
Forest plot showing the correlates of smoking cessation in men. (Legend) Forest plots of regression coefficients (95% Confidence Intervals) for the association of smoking cessation in men with socio-economic status (SES quintiles) (Reference = Q1), highest level of education (Reference = No formal education), employment (Reference = Not employed), marital status (Reference = Single), age, alcohol consumption (Reference = never consumed), smoking status (Reference = Never smoked). Model adjusted for site as a proxy for ethnicity. Estimates for men only (*N* = 2102)

Results suggest that the adjusted odds ratio (aOR) for smoking cessation increased with age, higher SES (4th and 5th quintiles), being married/living with partner, and having stopped drinking (Fig. 4). Current alcohol consumption was correlated with a lower likelihood of smoking cessation (Fig. 4; Supplementary Table 4).

## Discussion

In this study we reveal complex regional patterns of tobacco and alcohol consumption in six communities across four SSA countries. First, in the total cohort, almost half (49%) of all participants, men and women, either consumed tobacco products, alcohol, or both. Secondly, men were more likely to be current smokers than women (34.5% vs 2.1%), in line with global studies, but more women (14.4%) used smokeless tobacco (snuff and chewing tobacco) than men (5.3%), with some regional variation [2, 3, 5, 6]. Third, overall, being a current smoker was associated with alcohol consumption, lower education levels, and was less common among married individuals or those living with partners. Fourth, alcohol consumption was also more common among men, but had higher prevalence than smoking in both sexes (60.8% of men and 29.3% of women). And lastly, problematic alcohol use was associated with being male, widowed or divorced, and using tobacco products (smoking and smokeless use).

Prevalence estimates of current smoking among men from almost all the AWI-Gen sites were much higher when compared to the age-adjusted prevalence for daily smoking among men in their respective countries in a study conducted in 2015 [2]. A reason for this difference might be that the previous study to which we compare our findings was based on the prevalence of daily smoking, whereas our smoking variable was ‘current smoking’ with some men reporting that they were occasional current smokers. However, the direct comparison of prevalence of daily smokers is still higher in AWI-Gen and these differences could be due to several other factors including age distribution, and selection of specific communities in AWI-Gen, whereas Reitsma et al. (2017) may have used publications with small participant numbers, or specific co-morbidities from different communities to develop country-specific prevalence estimates. Patterns of tobacco use were highly sex-specific, with smoking tobacco most common among men, and women preferring snuff or chewing tobacco supporting previous findings in SA, Ethiopia, the Gambia, Kenya and Angola [3, 9, 10, 13, 15, 21, 25, 39]. In the combined sample, more than half of the men ever smoked, and 34.5% were current smokers. A similarly high prevalence of current smoking was previously reported in men from other African countries [3, 6, 9, 10, 13, 15, 21, 25, 39]. In our study, age was associated with current smoking in men (aOR = 0.98 (95%CI 0.97–0.99)) and smokeless tobacco consumption across both sexes (aOR = 1.06 (1.05–1.08)), and while statistically significant in the full dataset, the effects were small (per year of age) and in opposite directions. Adults in the highest wealth categories and those who had attained higher education levels were less likely to use tobacco products, in concordance with the findings of previous studies in Africa [3, 6, 9, 10, 13, 15, 21, 25, 39]. Alcohol consumption was strongly associated with tobacco use. Furthermore, former smokers appeared to be more likely to consume some form of smokeless tobacco, which suggests that there may be a substitution process at play. Smoking cessation was significantly associated with the highest SES quintiles, being married/living with a partner, and ceasing to consume alcohol. Within the AWI-Gen study, smoking was less common among adults with tertiary education and if they did smoke, they were more likely to stop smoking than those with lower levels of education.

Overall current alcohol consumption (both problematic and non-problematic) was present in 40.5% of the AWI-Gen cohort. The lower prevalence of alcohol consumption among women, as well as the decreased likelihood to engage in harmful alcohol consumption has been attributed to socio-cultural stigmas surrounding women who consume alcohol. This finding is supported by several studies in different African contexts [7–9, 17, 18, 20, 24]. Lifetime alcohol abstainers were more prevalent in East and South (Kenya and South Africa) Africa, compared to West Africa (Burkina Faso and Ghana). This difference was found to be more defined among women, with women in Nairobi (Kenya), Agincourt, Dikgale, and Soweto (South Africa) more likely to abstain from alcohol than participants in Navrongo (Ghana) and Nanoro (Burkina Faso). The frequency of daily alcohol consumption was highest among men in Nanoro (27.2%) and 10.3% of women were daily consumers. This trend of high daily alcohol consumption in both sexes was also observed during the national WHO-STEPS survey in 2013 in Nanoro where 26.2% of men and 16.7% of women were identified as daily alcohol consumers [36].

Differences in alcohol use were observed between the West African sites, Nanoro and Navrongo, despite having similar rates of alcohol consumption. Navrongo reported a much higher prevalence of problematic alcohol consumption (31.2%) than Nanoro (12.5%). In Navrongo, spirits were also more popular than in Nanoro. Binge drinking was found to be highest among men in Dikgale and Agincourt, corroborating previous data suggesting that SA has one of the highest rates of alcohol consumption per capita in the world [7, 17, 18, 40]. Binge drinking was the main feature of problematic alcohol consumption together with the feeling of needing to “cut down”. It also appears that social pressure through criticism (“people criticising you”) was most prevalent in Navrongo, followed by Dikgale. In Agincourt however, fewer surveyed drinkers reported facing criticism and feeling guilty related to their drinking patterns. In this study of adults aged 40 to 60 years, age was significantly associated with problematic alcohol consumption, where older people in this age range were less likely to drink, but the effect was small. Age effects related to drinking behaviour were observed in other African studies [12, 13, 16, 41]. Across the combined dataset, problematic drinking was not significantly correlated with SES, but SES associations were complex and varied at site level. A recent cross-sectional study examining correlates of alcohol use in the slums in Kenya suggested that alcohol use was associated with higher income, whereas the reverse relationship was found for problematic drinking in the Nairobi sample of this study (with lower odds of problematic drinking in association with higher SES) [10]. In our study, alcohol consumption was highly correlated with tobacco use, likely reflecting addictive behavior, rather than a preference for one substance above the other. This correlation has been observed in many cross-sectional studies that were also not specifically designed to reveal the likely sequence of substance use behaviours [9, 10, 13, 14, 16, 21, 39]. Current (problematic and non-problematic) and former drinkers were more likely to use tobacco products than those who had never consumed alcohol.

To our knowledge, this is one of the larger cross-sectional African population studies of multi-site comparisons of the prevalence of tobacco and alcohol consumption with related socio-demographic correlates. Patterns of tobacco and alcohol consumption show sex specific, regional (East, West, South Africa), as well as within-region differences. These differences may be due to differences in socio-economic transition across regions, but may also be affected by different national policies related to the regulation and taxation of tobacco and alcohol products, in turn affecting accessibility to those substances [2, 42, 43]. In addition, there are differences across religions and cultural beliefs which are known to influence patterns of tobacco and/or alcohol consumption [9, 10, 15, 17, 19, 24, 36].

### Limitations

This cross-sectional study was not designed to infer causality and there is no data to assess the temporal sequence of substance use. Our study was limited to persons aged 40 to 60 years, which is not representative of the general population, and data generated from a single community is not necessarily generalisable to be representative of the geographic region or an entire country. The missing data from Soweto limited inference for this site. Under-reporting of substance use may have occurred due to cultural differences within regions, as women would be less likely to report substance use due to potential stigmatisation, and because the CAGE questionnaire asks sensitive questions which participants may have found difficult to answer honestly and objectively.

## Conclusion

This study reports the prevalence and socio-demographic correlates of alcohol and tobacco consumption in four SSA countries (Burkina Faso, Ghana, Kenya, and South Africa). Our results showed differences in the prevalence of substance use, sex-differences in the type of tobacco products consumed, and the patterns of alcohol consumption across the study sites. Sex, SES, and education had varying effects on tobacco and alcohol consumption both regionally and by study site. The high prevalence of alcohol consumption and problematic alcohol use is of concern and policies should be strengthened on a macro level in order to combat this high prevalence of substance use in SSA [2, 42]. Individual behavioural changes may be more difficult to achieve without addressing them from a broader perspective, such as product-specific taxation, proactive monitoring and regulation, and limiting access to substances. Our results provide insight and understanding into patterns of tobacco and alcohol consumption in African communities in rural and urban settings, which is vital for the development of policies and interventions which may assist in reducing the burden of disease and mortality associated with substance use in Africa.

## Supplementary Information

**Additional file 1.**

**Additional file 2.**

**Additional file 3.**

**Additional file 4.**

## Data Availability

AWI-Gen phenotype data variables have been deposited in the European Genome-Phenome Archive (Accession number: EGAD00001006425). The datasets generated and/or analysed during the current study are available and accessible from the corresponding author Palwende Romuald Boua (romyboua@gmail.com) on reasonable request.

## Abbreviations

aOR: Adjusted odds ratio
AWI-Gen: Africa Wits-INDEPTH Partnership for Genomic Research Study
HDSS: Health and Demographic Surveillance System
HIV: Human Immunodeficiency Virus
LMIC: Low and middle income countries
Q1-Q5: First to fifth quintile for socio-economic status
QC: Quality control
SES: Socio-economic status
SA: South Africa
SSA: Sub-Saharan Africa
TB: Tuberculosis
